# Supplementary material for: Targeting mGlu1 Receptors in the Treatment of Motor and Cognitive Dysfunctions in Mice Modeling Type 1 Spinocerebellar Ataxia
Source: Cells. 2022 Dec 3;11(23):3916. doi: 10.3390/cells11233916 (PMC9738505; doi:10.3390/cells11233916)
Supplement: Supplementary file 1 [file cells-11-03916-s001.zip › cells-2070431-supplementary.pdf]

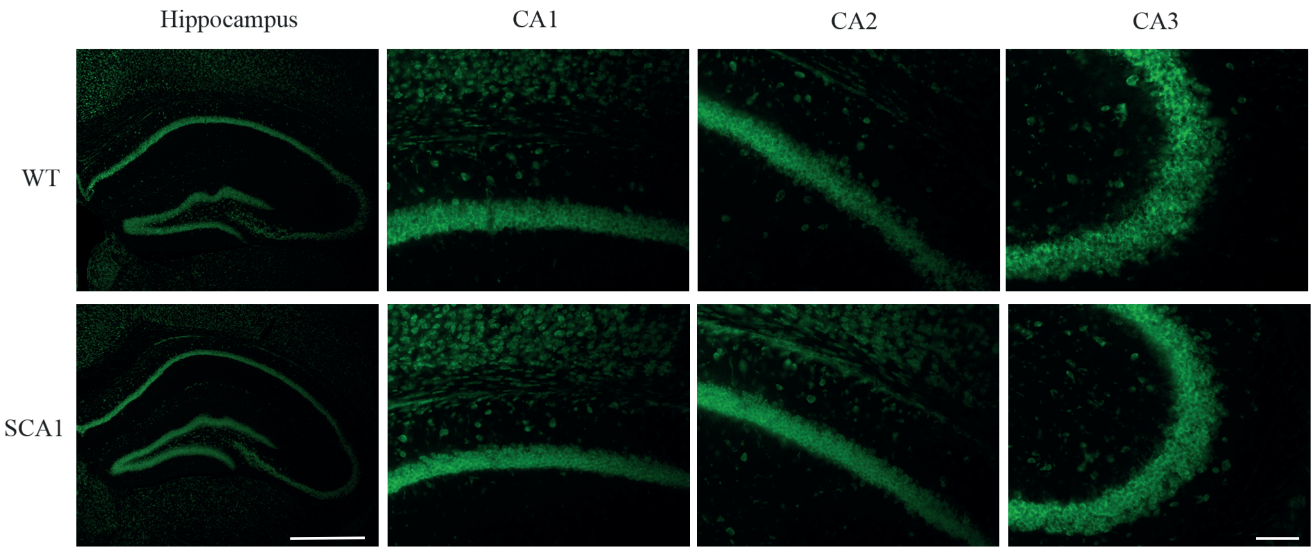

Figure S1: Representative image of Nissl staining in the hippocampus of WT and SCA1 mice.
Scale bar: 200 µm, 50 µm.

Figure S1
